# From Design to Prototype: High‐Sensitivity Tri‐Mode Operation Photodetectors Based on 1T‐2H Hybrid MoS_2_

**DOI:** 10.1002/advs.202520949

**Published:** 2026-01-28

**Authors:** Xinyu Li, Daxiu Tang, Jiaxin Guo, Xiao Zhang, Sihan Liu, Yaojun Yu, Qianqian Cheng, Chengwei Gao, Xiaoning Guan, Pengfei Lu, Fei Zhuge, Ying Xie, Changgui Lin, Xiang Shen, Haohai Yu, Huaijin Zhang, Jiyang Wang

**Affiliations:** ^1^ The Research Institute of Advanced Technology Ningbo University Ningbo China; ^2^ Digital Industry Research Institute Zhejiang Wanli University Ningbo China; ^3^ State Key Laboratory of Information Photonics and Optical Communications Beijing University of Posts and Telecommunications Beijing China; ^4^ Zhejiang SuperMat Sen‐Ray Optoelectronics Co., Ltd. Ningbo China; ^5^ Ningbo Institute of Materials Technology and Engineering Chinese Academy of Sciences Ningbo China; ^6^ State Key Laboratory of Crystal Materials and Institute of Crystal Materials Shandong University Jinan China

**Keywords:** 2D materials, capacitance effect, multi‐mode photodetectors, phase engineering

## Abstract

With the rapid progression of photoelectrical technology, the development of multi‐mode photodetectors is highly desirable for complex application scenarios. Here, we fabricate 1T‐2H mixed‐phase MoS_2_ films via electrochemical intercalation technique, which intrinsically integrates a 2H‐phase photosensitive network with a 1T‐phase dispersed capacitance. By modulating the external bias and phase distribution, a family of photodetectors with tailored tri‐mode operation was achieved among reconfigured photocapacitive (PCC), photovoltaic (PV), photoconductive (PC) modes. Breaking the conventional perception of capacitors as parasitic components in optoelectronic devices, our work harnessed the capacitive effect as an effective signal source, enabling self‐powered operation and high‐sensitivity detection. It exhibits efficient charging pulses and superior self‐resetting characteristics with a response speed reaching 19.2 ms and an average charge‐discharge efficiency of 87.6%. From device design to application prototype, our proof‐of‐concept demonstrations in non‐contact sensing, flame monitoring, and image reconstruction highlight the positive potential in intelligent sensing technologies. This study not only provides a novel design paradigm for phase engineering in multi‐functional photodetection, but also its successful implementations hold significant promising for advancing intelligent optoelectronics, such as optoelectronic chips and biosensing.

## Graphical abstract

This work demonstrates a phase‐engineered MoS_2_ photodetector that dynamically switches among photocapacitive (PCC), photovoltaic (PV), and photoconductive (PC) modes. By transforming the traditionally parasitic capacitance into an active signal source, the device achieves self‐powered, high‐sensitivity detection with a fast response of 19.2 ms, and a self‐resetting time of 21.3 ms enabling advanced applications in non contact sensing and image reconstruction.

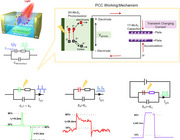

## 1 Introduction

With the rapid development of cutting‐edge artificial intelligence technologies, multi‐dimensional signal acquisition and processing capabilities have become the core competitiveness of next‐generation optoelectronic systems, whose typical applications cover dynamic environmental monitoring, multispectral sensing, human‐computer interaction, and other fields [1, 2]. Photodetectors, as the basic components of modern photonics technology, play an irreplaceable role in information recognition and conversion. Traditional photodetectors mainly rely on a single working mechanism, such as photoconductivity, photovoltaic effect, pyroelectric effect, etc. Their performance and functionality are always constrained by the photosensitive material properties (e.g., carrier mobility, complexity) and device architecture, which are unable to meet the higher demands in complex scenarios. Multi‐mode photodetectors have irreplaceable advantages in adaptive sensing under varying light intensities, self‐powered operation in power‐sensitive scenarios, and high‐speed transient detection requiring rapid response. These functions are difficult for traditional single‐mode operation detectors to achieve comprehensively. Currently, there are related research reports on dual‐mode detection based on heterostructures, such as the dual‐mode photoresponse detection of Gr‐MoS_2_‐VO_2_ composite structure [3] and the dual‐mode photodetection of MoTe_2_‐MoS_2_ heterojunction [4]. However, the limitations of traditional single‐mode detectors are particularly prominent: pure PC mode detectors require continuous external bias and cannot function in passive sensing scenarios; pure PV mode detectors have slow response speeds and are difficult to capture transient signals; pure PCC mode detectors have rapid signal attenuation and cannot achieve long‐term steady‐state monitoring. Therefore, there is an urgent need for devices with multiple working modes and dynamic switching mechanisms [5, 6, 7, 8, 9, 10].

Molybdenum disulfide (MoS_2_), a 2D layered material, offers unprecedented candidates for high‐performance photodetectors due to its impressive properties, such as atomic‐level thickness, diverse phase structure, tunable electronic structure, and strong light‐matter interactions [11, 12, 13, 14, 15]. Most of the existing MoS_2_‐based detectors are still confined to the photoconductivity effect mode due to the inefficient carrier control at heterogeneous interfaces [16]. Fortunately, the strong synergy between the semiconducting 2H‐phase MoS_2_ and the metallic 1T‐phase MoS_2_ has been explored as an effective strategy to optimize their photoelectronic properties. It has been proved the former 2H‐phase MoS_2_ ensures the strong light absorption capability, and the latter 1T‐phase MoS_2_ can promotes the efficient charge transport [17, 18, 19, 20]. More importantly, the spatial separation and dynamic storage of photogenerated charges can be realized by precisely tuning the energy band arrangement and carrier transport paths through phase transition engineering [21, 22, 23]. For example, Wang et al. observed the bi‐directional migration and selective storage of photogenerated carriers at the 1T'/2H heterojunction interface, showing the effectiveness of phase engineering for charge regulation [19]. Tang et al. showed that the 1T/2H MoS_2_ Schottky homojunction‐based bulk heterojunction realizes 380–900 nm broadband photovoltaic effect via built‐in electric field from 1T/2H band gap difference, achieving self‐powered photodetection with excellent performance at zero bias [24]. However, as the heterostructure is thinned down to the nanoscale, capacitance effects (especially parasitic capacitance) are often accompanied by non‐negligible problems such as signal attenuation, noise interference, and response delay, which are particularly prominent in low‐light and transient detection scenarios. Recent studies have shown the synergistic optimization of capacitance effects in novel non‐contact measurements. The dual‐mode non‐contact capacitive sensor developed by Huang et al. exhibits distinct advantages, including ultra‐fast dynamic response and robust anti‐interference capability [25]. Ahn et al. achieved synergistic optimization of parasitic capacitance suppression and carrier modulation through phase engineering, thereby further improving the device's signal‐to‐noise ratio [26]. So far, its positive utilization for functional photodetection is still little‐researched.

Here, we demonstrate a family of novel multifunctional photodetectors based on ultra‐thin mixed‐phase MoS_2_ films equipped with spatially separated functional region: the metallic 1T phase acts as capacitive‐sensitive and the semiconducting 2H phase conductive‐dominant regions. Through heterojunction interface engineering and external field co‐regulation, the device exhibits capacitive‐conductive‐photovoltaic tri‐modal operation and achieves dynamically programmable switching. Leveraging its excellent sensitivity to light intensity variations, our device demonstrates versatile applicability from design to prototyping in non‐contact measurement, flame monitoring, and image reconstruction. These successful implementations hold significant promising for next‐generation intelligent and collaborative technologies.

## 2 Results and Discussion

**FIGURE 1:**
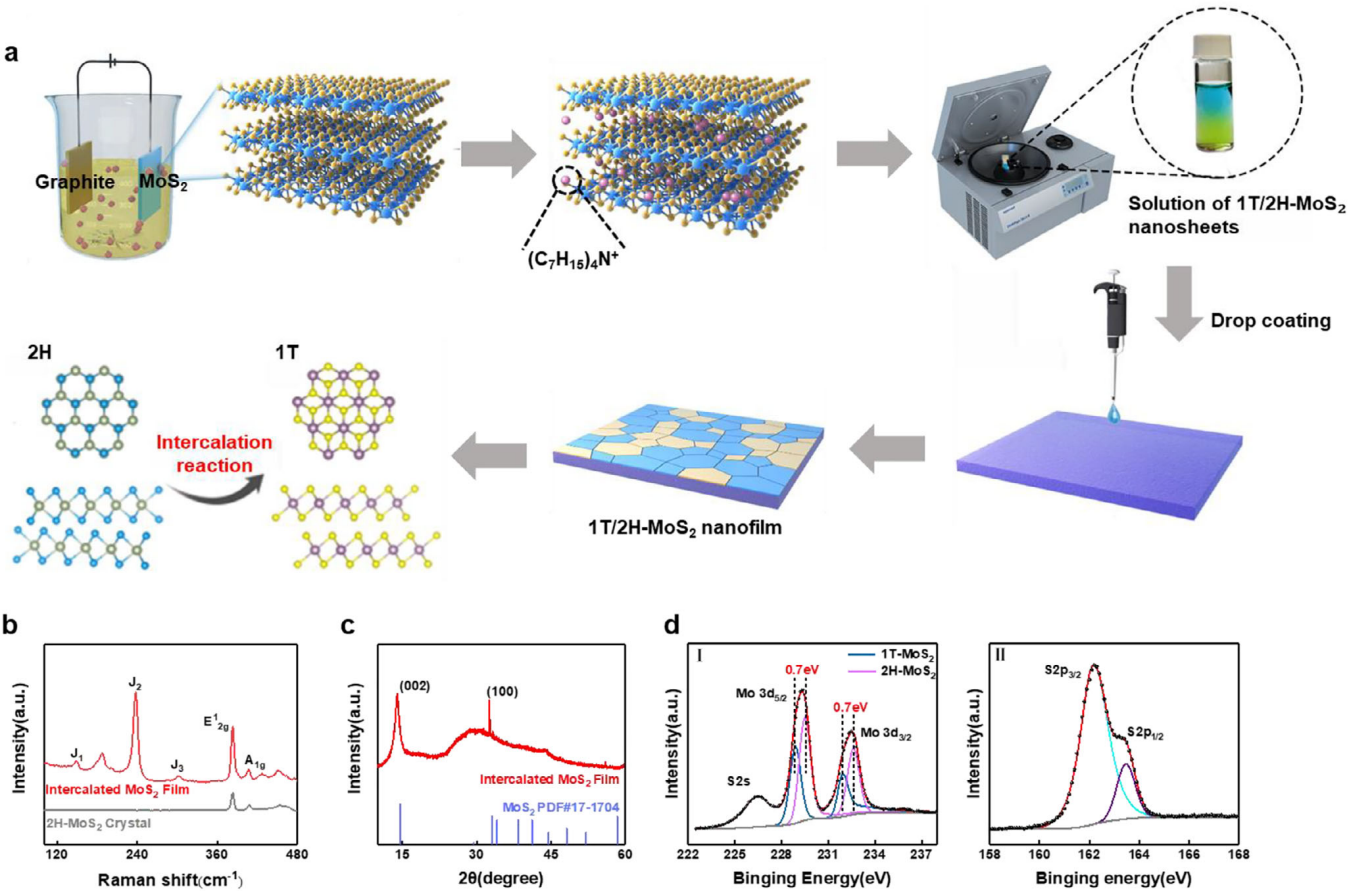
(a) Preparation process of MoS_2_ nanosheets and schematic diagram of the phase structure of 1T/2H MoS_2_ (b) Raman spectra of MoS_2_ (c) X‐ray diffraction patterns of MoS_2_ thin films (d) XPS patterns of elemental Mo S for I and II, respectively.

The mixed‐phase MoS_2_ films were prepared using electrochemical intercalation technology, as shown in Figure 1a. This technology achieves exfoliation by inserting large‐sized ions into the van der Waals gaps of bulk MoS_2_. Subsequently, the nanosheet solution after centrifugal cleaning was dropped onto the silicon substrate to form a uniformly distributed film. The detailed preparation process refers to the Note S1. Three kinds of MoS_2_ film devices with different thicknesses were fabricated, which were characterized to be 23, 31, and 50 nm via atomic force microscopy (AFM) in Figure S1. Scanning electron microscopy (Figure S2) observed a large number of irregularly arranged MoS_2_ fragments distributed on the sample surface, showing a complex stacking state. To investigate the surface structure of the samples, the Raman spectra of the MoS_2_ bulk crystals and the intercalated thin films were tested (Figure 1b): both showed characteristic peaks at 383.2 and 406.8 cm^−1^, corresponding to in‐plane (E ^1^_2 g_) and out‐of‐plane (A_1g_) vibrational modes. The intercalated films also showed characteristic J_1_, J_2_, E_1g_ and J_3_ peaks of 1T‐MoS_2_ at 148.4, 187.7, 237.4 and 301.6 cm^−1^ [27]. The Raman spectra and the analyzed phase compositions of different thicknesses are detailed in Figure S3. The results showed a similar 1T/2H ratio across the thin and thick film. The comparative Raman patterns indicate that the method successfully synthesized hybrid films of 1T‐ MoS_2_ and 2H‐ MoS_2_, and the phase transition from 2H to 1T occurred during the preparation. Comparison of the X‐ray diffractograms (Figure 1c) of the intercalated MoS_2_ films with the PDF#17‐1704 standard card shows that 2*θ* equals to 13.9^°^ and 32.6^°^ corresponds to the (002) and (100) crystal planes, respectively. A significant broadening of the half‐height width (about 13.9^°^) was observed, which may be attributed to the overlap of peaks due to the difference in the position of the (002) crystallographic phase of 1T‐ MoS_2_ and 2H‐ MoS_2_ [28]. X‐ray Photoelectron Spectroscopy (XPS) (Figure 1d) showed that there are two pairs of double peaks with a binding shift of 0.7 eV. Therein peaks at 232.7 and 229.5 eV corresponded to the 2H phases Mo3d_3/2_ and Mo3d_5/2_, while the peaks at 231.9 and 228.8 eV were attributed to the 1T‐MoS_2_, which is agree with previous reports and further confirms the coexistence of the 1T and 2H mixed‐phases [29]. Besides, the effectiveness of intercalant removal was confirmed by Fourier‐transform infrared spectroscopy (FTIR) and characteristic peaks of organic substances in XPS, as detailed in the (Figure S4).

**FIGURE 2:**
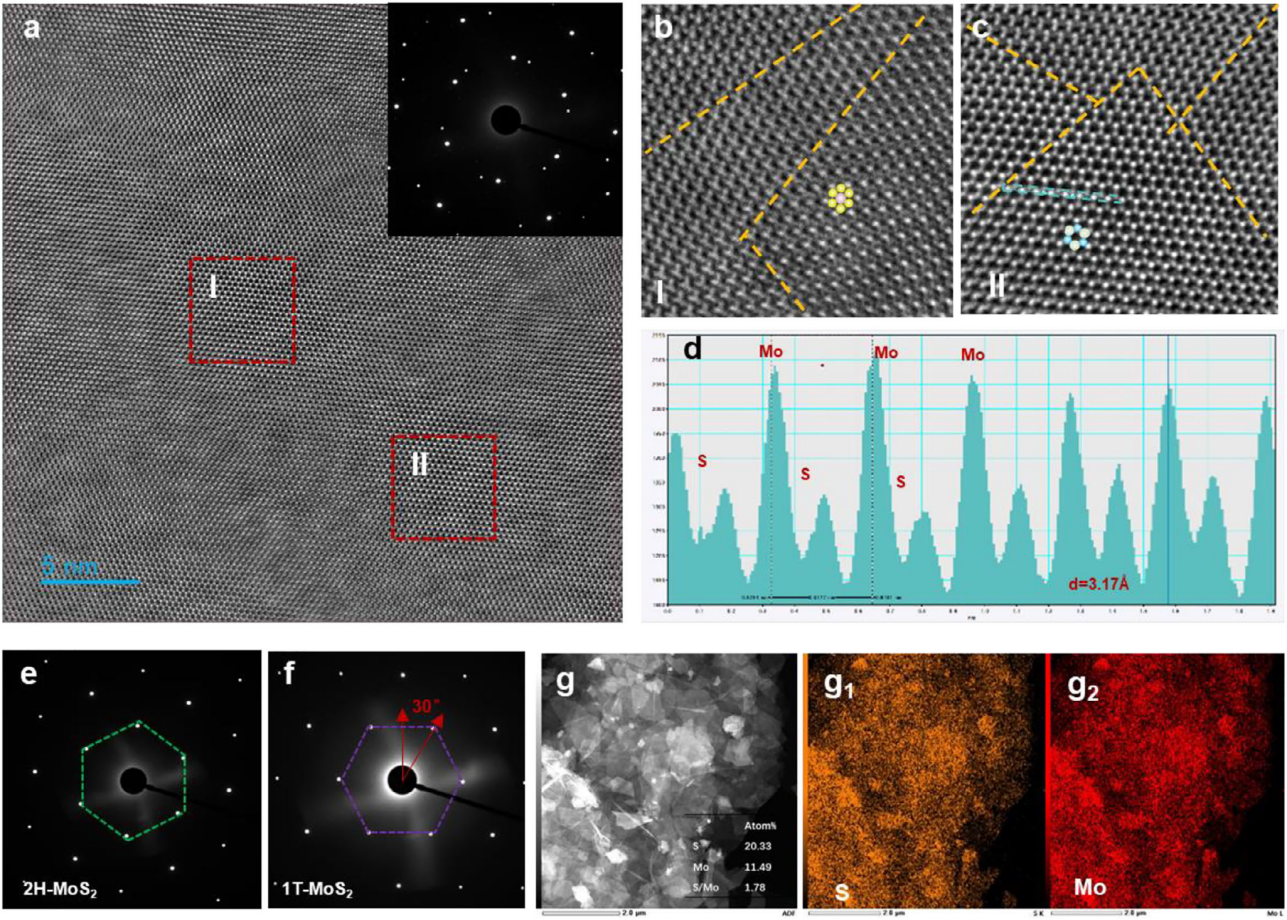
(a) HRTEM image of the MoS_2_ film and the corresponding SAED pattern. (b,c) Magnified HRTEM images corresponding to the red‐boxed region in (a). (d) Lattice fringe analysis corresponding to the blue line in (c), showing an interplanar spacing of 3.17 Å. (e,f) SAED patterns collected from isolated 1T and 2H phase regions. (g) The characteristic stacked nanolayer morphology and EDS spectrum of MoS_2_ film.

The high‐resolution transmission electron microscopy (HRTEM) analyses were performed for in‐depth insight into the crystallinity and lattice structure of as‐prepared MoS_2_ thin film. The low‐magnification HRTEM image in Figure 2a shows an atomically sharp lattice and the corresponding selected‐area electron diffraction (SAED) pattern displays clear diffraction spots, confirming the high crystallinity of the film. Notably, the presence of multiple sets of symmetrically arranged diffraction spots indicates the coexistence of multiple phases. The boundaries between different phases or crystal planes are highlighted by yellow lines in the enlarged lattice micrographs (Figure 2b,c), directly demonstrating the presence of regions with distinct atomic arrangements. And the alternating stacking arrangement of Mo and S atoms shows the lattice spacing was 3.17 Å (Figure 2d), which is in good agree with the reported MoS_2_ sandwich structure with standard lattice space of 3.16 Å [30]. Furthermore, SAED patterns were collected from isolated 1T‐phase regions (Figure 2e) and 2H‐phase regions (Figure 2f) to independently confirm the monocrystalline nature of each phase. Both patterns exhibit well‐defined, single‐crystal hexagonal spot arrays and both display the 30° rotational characteristic, strongly supporting that the well‐crystallized 1T and 2H phases are randomly intermixed within the thin film [31]. The energy‐dispersive X‐ray spectroscopy (EDS) profile shown in Figure 2g demonstrates a high degree of uniformity in the spatial distribution of Mo and S elements, confirming the successful synthesis with excellent compositional homogeneity.

**FIGURE 3:**
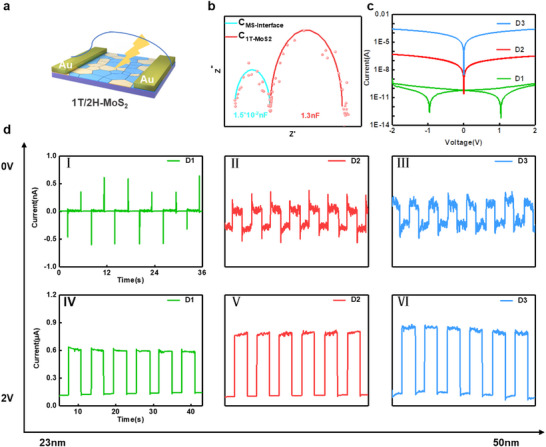
(a) Schematic diagram of MoS_2_ detector. (b) Impedance spectra at different positions inside the MoS_2_ photodetector. (c) *I*–*V* characteristics of MoS_2_ photodetectors with different thicknesses (d) *I*–*T* characteristics of MoS_2_ photodetectors under 532 nm incident light with a power of 45 µW. (I, II, III) *I*–*T* characteristics of MoS_2_ detectors with thicknesses of D1, D2, and D3 at 0 V; (IV, V, VI) *I*—*T* characteristics of MoS_2_ detectors with thicknesses of D1, D2, and D3 at 2 V.

To investigate the influence of the hybrid phase on the optoelectronic properties of molybdenum disulfide (MoS_2_) materials, prototype 1T/2H‐phase MoS_2_ photodetectors with thicknesses of 23, 31, and 50 nm (corresponding to D1, D2, and D3 respectively) were constructed by depositing gold (Au) electrodes on the sample surface using an ion sputtering apparatus (Figure 3a). The current–voltage (*I*–*V*) characteristic curves in the dark state were shown in Figure 2c, exhibiting a high degree of symmetry, which confirms the ohmic contact between the electrode and the film. However, it is noteworthy that the bidirectional scanning *I*–*V* characteristic curves of the D1 device shows a significant current hysteresis (shown by the green curve in Figure 3c), which is indicative of a typical capacitive feature. This phenomenon indicates that the capacitive effect within the device is non‐negligible, which is generally attributed to the relaxation effect from the distributed capacitive network of stacked nanosheets. In contrast, D2 and D3 exhibited conventional semiconductor conduction behavior. Corresponding Nyquist impedance spectra, as showed in Figure S5, effectively confirmed the nanoscale stacking state and induced interfacial effects of the 1T‐phase nanosheets is governed the capacitive effect and response mode. It may be because the film is thicker, the 1T‐phase nanosheets are stacked more densely, which generated a smoother charge transport and weakened capacitive effect. Further Nyquist impedance spectrum (Figure 3b) of D1 is combined with the ZView software equivalent circuit building simulation to quantitatively determine the size of the capacitance region. It is obvious that a double relaxation semicircle model matches and the capacitance value are calculated by using the formula 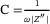 (*ω* is the angular frequency and *Z″* is the imaginary part of impedance at the corresponding frequency) to be *C* = 1.5×10^−2^ nF and 1.3 nF. The smaller semicircle is attributed to the contact interface capacitance between the gold electrode and the film (*C_MS‐Interface_*), while the larger semicircle originates from the distributed capacitance formed by the 1T phase nanosheet stack (*C_1T‐MoS2_*). The results agree well with the *I*–*V* measurement.

The photocurrent‐time (*I*–*T*) characteristics were measured of three devices to further explore dynamic photoresponse mechanisms. Under the co‐regulated of bias voltage and film thickness, it reveals three distinct response modes, as shown in Figure 3d. The responsivity of the device can reach up to 15.6 mA/W. The spike‐pulse signal in Curve I originates from the capacitive effect. Curves II and III exhibit square‐wave signals under zero external bias, corresponding to the photovoltaic (PV) response originated from 1T/2H heterojunction. Notably, Curve II simultaneously displays both spike‐pulse and square‐wave features—illustrating the dynamic switching process between capacitive and PV‐dominated mechanisms. The retained small transient spikes are attributed to the instantaneous charging of residual nano‐capacitors at sparse 1T‐2H interfaces, rather than thermal effect, with relevant explanations provided in Figure S6.

When a bias voltage of 2 V is applied, all films (Curves IV–VI) show square‐wave signals, dominated by the photoconductive (PC) mechanism. The gradual increase in photocurrent arises from enhanced light absorption, and the corresponding absorption spectrum is provided in Figure S7. The absorption spectrum shows that light absorption is significantly enhanced with increasing film thickness, implying that the photoelectric conversion capability is improved. Overall, the device enables switching between PCC and PC, by tuning the bias voltage, and between PCC and PV by adjusting the film thickness.

**FIGURE 4:**
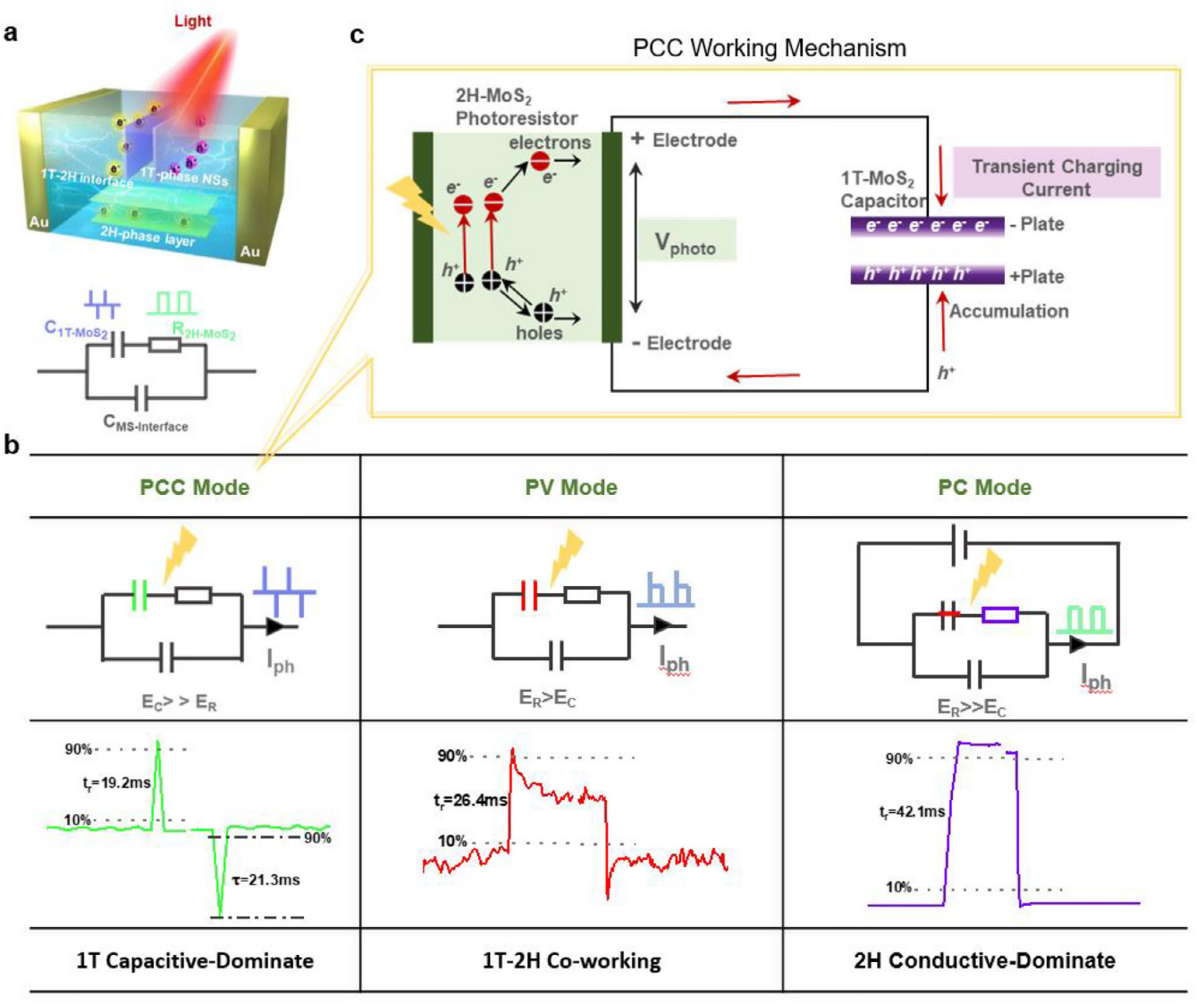
(a) Schematic of the basic building block and simplified equivalent circuit of the device in the dark state (b) Simplified circuits and response times of the three operating modes of the device (PCC: 19.2 ms, PV: 26.4 ms, PC:42.1 ms) (c) Analysis of the transient working mechanism of the PCC mode.

Based on the above analysis, the device can be simplified as a 3D series‐parallel network formed by the random connection of in‐plane 2H photoconductors and interlayer 1T capacitors. Figure 4a schematically illustrates the basic building block. Within this system, 2H‐phase layer functions to generate and transport photogenerated carriers, while the stacked face‐to‐face 1T‐phase nanosheets inherently form microscopic parallel‐plate capacitors with the capacity for charge store and release. This unique dual‐phase structure inevitably contains 1T‐2H heterojunction interfaces, which also serve as the fundamental basis for the photovoltaic effect. The corresponding simplified equivalent circuit of the device under the dark state is shown in Figure 4a for understanding the synergy and competition between these components in the device's multi‐mode operation. It clearly distinguishes the synergistic relationship between the interface capacitance (*C_MS‐Interface_)*, the distributed structure capacitance (*C_1T‐MoS2_*), and the body resistance (*R_2H‐MoS2_*), and provides a theoretical basis for the dynamic switching behavior. According to Figure 4, the device exhibits three distinct operational modes, governed by external bias and internal structure, each with a unique working principle and performance characteristics. Their respective working mechanisms and simplified circuits are both summarized in Figure 4b. For D1, under zero external bias, the device operates in a self‐driven, 1T capacitive‐dominant mode (PCC mode). The 2H‐phase semiconductor regions generate a photovoltage (*V_ph_*) via the photovoltaic effect at the 2H/1T heterojunction, charging the distributed network of 1T nano‐capacitors. Upon the removal of light, these capacitors discharge through external circuits. The illustration of PCC mode is presented in Figure 4c. The transient charging current originates from the synergistic effects of photoconductive carrier generation within the 2H phase and subsequent interface‐driven carrier separation at the 1T‐2H junctions. This process first leads to a rapid carrier accumulation at the interfacial trapping sites during the transient‐state, resulting in the observed current spike (PCC mode). As the illumination continues, this charging rate diminishes, and the system reaches an equilibrium where the generation rate balances the recombination/transport rate, leading to a steady, lower current flow characteristic of the steady‐state (PV or PC mode).

At this moment, the capacitive effect in the circuit is far stronger than the resistive effect (E_C_ >> E_R_), leading to a typical capacitive pulse spike signal with a response speed (t_r_) of 19.2 ms (corresponding to a calculated −3 dB bandwidth of approximately 18.2 Hz) and a self‐resetting time (τ) of 21.3 ms. With increased film thickness (e.g., D2), a hybrid capacitive‐ photovotailc mode (PV) emerges. In this state, the photovoltage from the 2H‐phase and the capacitive charging effect of the 1T‐phase cowork and contribute jointly to the photoresponse (*E_R_* > *E_C_*). The conductive percolation network from the thicker 1T phase also facilitates a stable photocurrent component. This combined mechanism results in a response speed of 26.4 ms, corresponding to a calculated −3 dB bandwidth of approximately 13.3 Hz. When an external bias voltage (*V_bias_* ≥ 2 V) is applied, the 1T capacitive elements are electrically broken down and shunted (*E_R_* >> *E_C_*). This renders the capacitive network ineffective and switches the device into a purely photoconductive mode (PC mode). The photoresponse is now governed solely by the bandgap excitation in the 2H‐phase semiconducting regions and the subsequent drift of carriers under the external electric field. Compared with the capacitance‐involved response, a stable photocurrent with a response speed of 42.1 ms was detected, which corresponds to −3 dB bandwidth of about 8.3 Hz.

**FIGURE 5:**
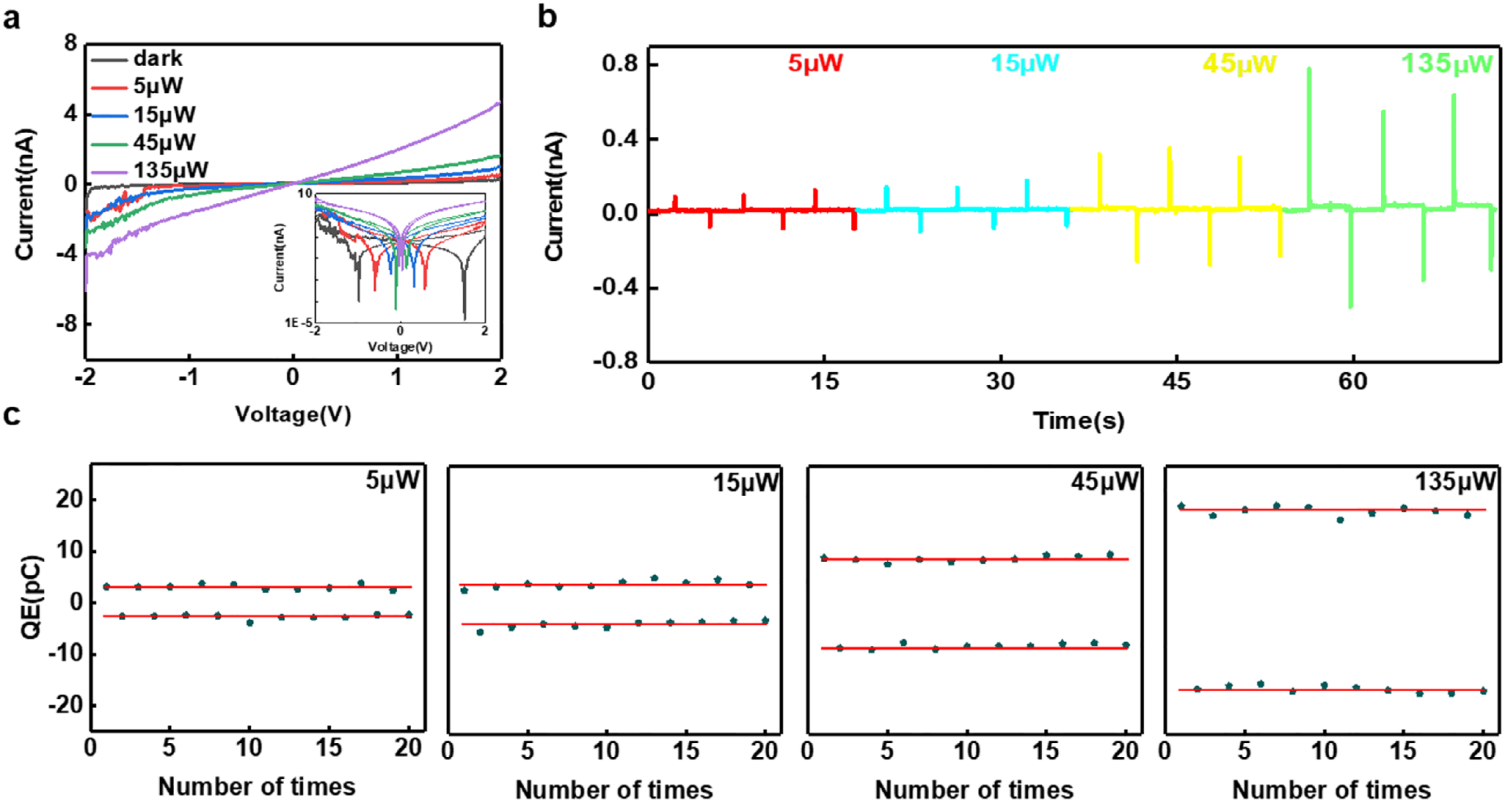
(a) *I*–*V* characteristics of MoS_2_ photodetectors (b) *I*–*T* characteristics of MoS_2_ photodetectors with different light intensities (c) Capacitance charge variations with different light intensities.

Figure 5 further explores the relationship between the capacitance‐effect‐dominated photoresponse and light intensity under 532 nm laser irradiation. *I*–*V* curves (Figure 5a) show the photocurrent increases nonlinearly with light intensity, indicating the excellent detection sensitivity to light intensity. The inset bidirectional scanning *I*–*V* characteristic curves exhibits the same hysteresis phenomenon as Figure 3c. Meanwhile, the hysteresis observed gradually diminishes under illumination. It may be due to the 1T phase provides the maximum charge accumulation capacity in the dark state and as the light intensity increases, the charge accumulation sites formed by photogenerated carriers at the 1T‐2H interface approach saturation gradually. When the light is illuminated, the photogenerated voltage drives the charge to migrate rapidly into the capacitive region formed by the 1T‐MoS_2_ nanosheets, triggering a positive spike current; after the light is turned off, the stored charge is released rapidly, generating a negative spike current. The experimental *I*–*T* curves (Figure 5b) show that the spike current amplitude increases with the light intensity from 0.1 nA at 5 µW to 0.7 nA at 135 µW, and the stored charge of the capacitor calculated according to $\int_{0}^{t}\cdot \left(t\right)dt$ (where *t* denotes the charging or discharging time and *i(t)* is the current during that time interval) increases synchronously from 3 to 18 pC, which confirms the efficient charging/discharging process of the flat plate capacitor (*C_1T‐MoS2_* = 1.3 nF) driven by the photogenerated voltage. Figure 5c further reveals the periodic oscillatory characteristics of the capacitor charge state: rapid accumulation of charge to the peak value (*Q_max_* = 18.5 pC) in the light phase and rapid release of charge to complete the discharge in the dark phase. The charge‐discharge efficiency ($\eta =\frac{Q_{\mathrm{discharge}}}{Q_{\mathrm{charge}}}$) is 87.6% ± 1.2 % (standard deviation) from three parallel tests. The Q_charge_ values are 17.8 pC, 18.5 pC, 18.2 pC, and Q_discharge_ values are 15.6 pC, 16.2 pC, 15.9 pC. All deviations are < 3%, verifying test data reliability. These characteristics highlight its potential for important applications in the fields of information processing and fast‐change signal acquisition. The key performance metrics of MoS_2_ photodetectors has been summarized in Table S1, highlighting the advantages in multi‐mode operation and high energy utilization efficiency.

**FIGURE 6:**
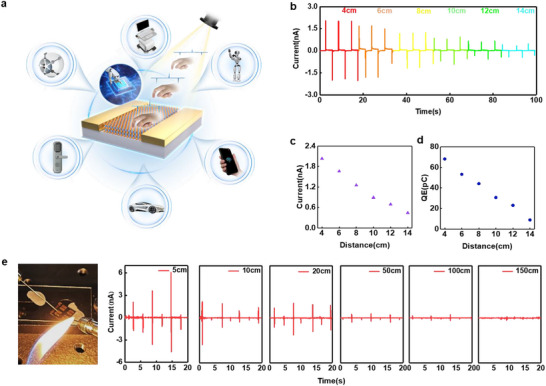
(a) Schematic diagram of a non‐contact sensor (b) Variation of current with distance under ambient light (c) Average current vs. distance (d) Capacitive charge vs. distance (e) Schematic diagram of a fire detection scenario and characteristic curves of current vs. flame distance.

Therefore, taking advantage of its high acuteness to light intensity variations, we evaluated its performance in non‐contact measurement scenarios in advanced intelligent devices, for example in Figure 6a. As shown in Figure 6b, under ambient white light conditions, as the distance between the external object and photodetector increases from 4 to 14 cm via an electrically precise displacement stage, the spiked photocurrent decreases gradually. As shown in Figure 6c, the photocurrent decreases from the initial 2.05 to 0.45 nA with an approximately linear distribution. The charge value of the capacitor during charging and discharging is calculated using the equation $\int_{0}^{t}\cdot i\left(t\right)dt$. As shown in Figure 6d, the calculated capacitor charging and discharging values decrease from 68 to 8 pC in an approximately linear trend, confirming the sensitivity to distance variation. Furthermore, the device was tested using an alcohol lamp flame as a non‐conventional light source to simulate a practical alarm scenario (Figure 6e). The signal intensified when the flame grew stronger or the detector was moved closer, accurately reflecting the fire's severity and location. Conversely, the signal weakened or even reversed when the flame weakened or the detector was pulled away, enabling real‐time monitoring of the fire's status. This validates the device's effective response in real‐world scenarios and its potential to significantly improve the efficiency and accuracy of fire prevention and response systems. Overall, the device demonstrates cutting‐edge performance in non‐contact measurement with high sensitivity and fast response, showing application potential in biomedicine, human‐machine interface, and medical devices, driving the development of smart devices towards human‐machine collaboration.

**FIGURE 7:**
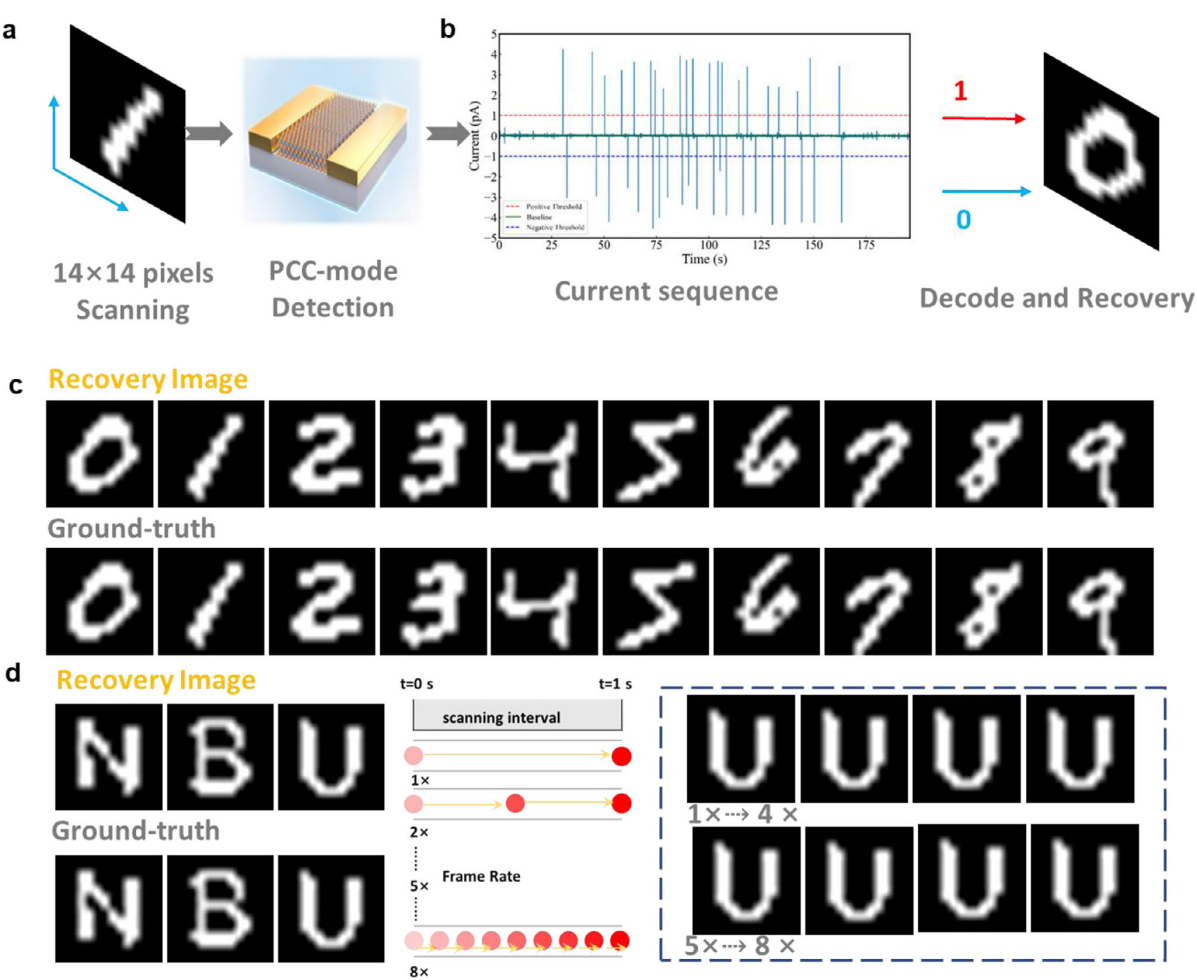
(a) Schematic diagram of the digital reconstruction experiment configuration (b) Time‐domain current sequence diagram corresponding to the number "0" (c) High‐fidelity reconstruction image of the numbers 0 to 9 (d) High‐fidelity reconstruction image of the letters N, B, U, and the letter U at different construction speeds.

Leveraging the fast, self‐resetting, and event‐triggered pulse characteristics observed in the PCC mode, the potential application for next‐generation computational vision was recognized, particularly mimicking the operational principles of event cameras. As shown in Figure 7, the image information reconstruction experiment was designed to validate the device's unique advantage in data encoding and advanced information processing. Two groups of reconstruction experiments were conducted using ten numerical binary digits (0–9) and three letters (N, B, and U) from MNIST database as target patterns [32]. The experimental configuration is schematically illustrated in Figure 7a. Each binary target (14 × 14 pixels) was discretized into 196 individual point sources and the temporal photocurrent signal of the detector was acquired point‐by‐point. Specifically, the detector generates a positive photocurrent pulse when a pixel transitions from 0 to 1, whereas a negative current response was induced upon the 1→0 pixel transition. Figure 7b displays the temporal current sequence corresponding to digit “0”. By defining appropriate thresholds for positive and negative current values, the original spatial information of the target was successfully decoded from the time‐domain photocurrent signals. High‐fidelity reconstructed images of the numerical digits and letters are displayed in Figure 7c,d, respectively. These results explicitly demonstrate the detector's superior sensing and information reproduction capability across diverse target patterns. To further assess its robustness under dynamic conditions, additional reconstruction tests were performed at eight distinct scanning rates (corresponding to frame rates of 1×, 2×, 3×, 4×, 5×, 6×, 7×, and 8× the base rate). Notably, consistent high‐fidelity reconstructions were achieved across all eight frame rate conditions. This outcome corroborates that the proposed detector maintains reliable target capture and information decoding performance even when subjected to varying dynamic scanning demands.

## 3 Conclusion

In summary, we prepared 1T‐2H mixed‐phase MoS_2_ films by electrochemical intercalation combined with drop coating. The film integrates a 1T phase distributed capacitance network and a 2H phase light‐absorbing region. By adjusting the external bias voltage and phase distribution via phase engineering, the based photodetector exhibits operational switching among three PCC, PC, and PV modes and achieves excellent photoelectric performance with the photoresponsivity reaching 15.6 mA/W, response speed of 19.2 ms, and a self‐resetting time of 21.3 ms in the visible spectrum. Notably, the device converts the capacitive effect into a detection advantage through phase engineering, overcoming the traditional notion that “parasitic capacitance is interference”. It shows unique advantages such as high sensitivity detection, efficient charge storage/release, and self‐resetting without external bias. In addition, our prototyping demonstrations in non‐contact sensing, flame monitoring and image reconstruction demonstrate its versatility and stability. This work provides a brand‐new design concept for reconfigurable multi‐mode photodetectors, laying a foundation for the development of intelligent sensing technology in fields such as biosensing, unmanned vehicles, and human‐computer interaction.

## Conflicts of Interest

The authors declare no conflicts of interest.

## Supporting information




**Supporting File**: advs74134‐sup‐0001‐SuppMat.docx.

## Data Availability

The data that support the findings of this study are available from the corresponding author upon reasonable request.
