# Supplementary material for: From Design to Prototype: High‐Sensitivity Tri‐Mode Operation Photodetectors Based on 1T‐2H Hybrid MoS2
Source: Adv Sci (Weinh). 2026 Jan 28;13(20):e20949. doi: 10.1002/advs.202520949 (PMC13067817; doi:10.1002/advs.202520949)
Supplement: Supplementary file 1 — Supporting File: advs74134‐sup‐0001‐SuppMat.docx. [file ADVS-13-e20949-s001.docx]

***Supporting Information* of**

**From Design to Prototype: High-Sensitivity Tri-mode Operation Photodetectors Based on 1T-2H Hybrid MoS_2_**

*Xinyu Li^1^, Daxiu Tang^2^, Jiaxin Guo^1^, Xiao Zhang^1^, Sihan Liu^1^, Yaojun Yu^1^, Qianqian Cheng^1^*, Chengwei Gao^1^, Xiaoning Guan^3,4^, Pengfei Lu^3,4^, Fei Zhuge^5^**, Ying Xie^1^*, Changgui Lin^1^, Xiang Shen^1^, Haohai Yu^6^, Huaijin Zhang^6^, Jiyang Wang^6^*

1 The Research Institute of Advanced Technology, Ningbo University, Ningbo, 315211, China

2 Digital Industry Research Institute, Zhejiang Wanli University，Ningbo, 315100, China

3 State Key Laboratory of Information Photonics and Optical Communications, Beijing University of Posts and Telecommunications, Beijing, 100876, China

4 Zhejiang SuperMat Sen-Ray Optoelectronics Co., Ltd., Ningbo 315400, China

5 Ningbo Institute of Materials Technology and Engineering, Chinese Academy of Sciences, Ningbo, 315201 China

6 State Key Laboratory of Crystal Materials and Institute of Crystal Materials, Shandong University, Jinan 250100, China

**Keywords:** two-dimensional materials; multi-mode photodetectors; capacitance effect; phase engineering

**Supporting Information** **Note 1：**

The intercalation technique is carried out at room temperature using a two-electrode system, with bulk molybdenum disulfide (MoS_2_) crystals as the cathode and a graphite rod as the anode. The electrolyte is a 60 mL acetonitrile solution of tetraheptylammonium bromide (THAB) at a concentration of 10 mg/mL. A constant voltage of -10 V is applied to the cathode (with a current of approximately 30 mA) for 0.5 hours, driving the tetraheptylammonium cations (THA^+^) in the electrolyte to intercalate into the MoS_2_ layers, causing lattice expansion. Subsequently, the intercalated MoS₂ is placed in a 60 mL 0.2 M dimethylformamide (DMF) solution and subjected to 1 hour of ultrasonic treatment, successfully exfoliating it into a deep green and uniform nanosheet dispersion. To obtain pure nanosheets, the dispersion is subjected to gradient centrifugation: first, it is centrifuged at 2000 rpm for 5 minutes to remove unexfoliated thick flakes as sediment, and then the supernatant is collected and centrifuged again at 9000 rpm for 20 minutes. The resulting sediment is washed three times with isopropanol to remove the reaction by-products and residual intercalants and finally redispersed in isopropanol. By drop-casting this purified nanosheet solution onto a silicon substrate and drying it at room temperature, a uniformly distributed MoS₂ film can be formed.


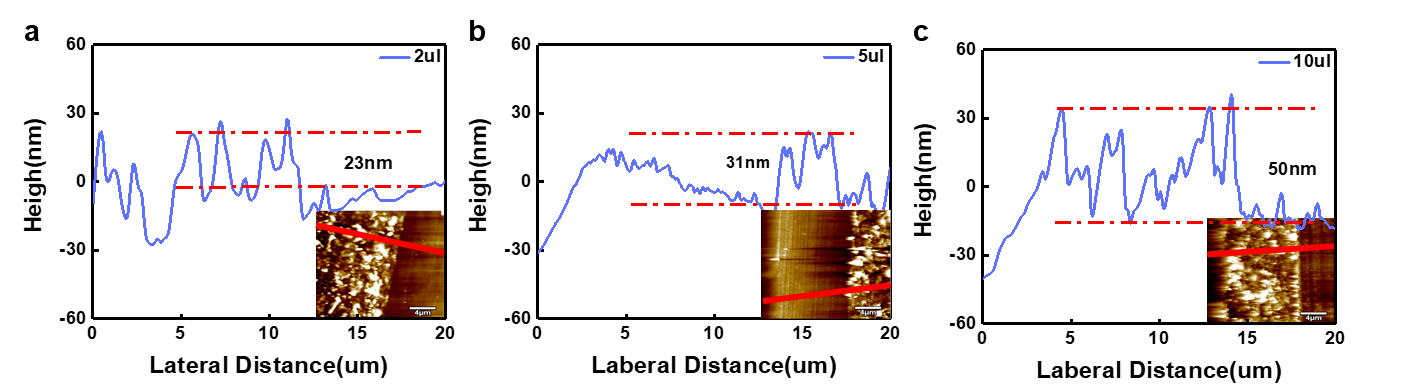


Figure S1: (a), (b), and (c) show the thicknesses of MoS_2_ films at the base map volumes of 2 μL, 5 μL, and 10 μL, respectively

To obtain the correlation between the drop-coating solution dose and the thickness of the nanosheet film, atomic force microscopy (AFM) was used for the analysis. The film thicknesses of 2 μL, 5 μL, and 10 μL of drop-coated solution on the surface of the silicon substrate are shown in Figure S1. a-c, respectively, and the insets are the corresponding surface topography images. The film thicknesses were measured to be 23 nm, 31 nm, and 50 nm, respectively, which indicate a significant positive correlation between the drop-coating solution dose and the sample thickness.


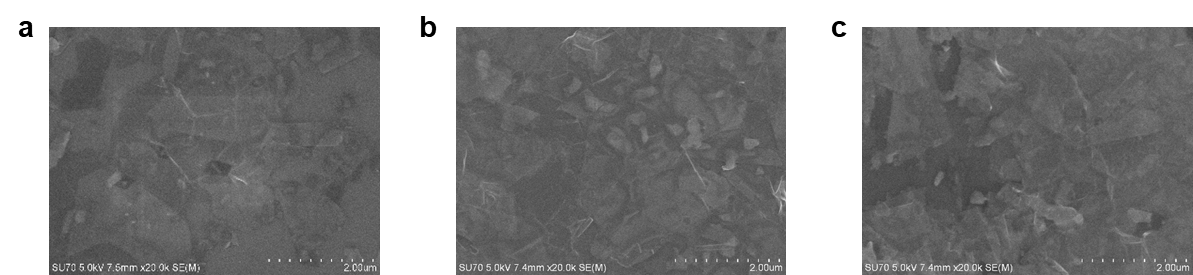


Figure S2: The surface morphologies of MoS_2_ with different thicknesses


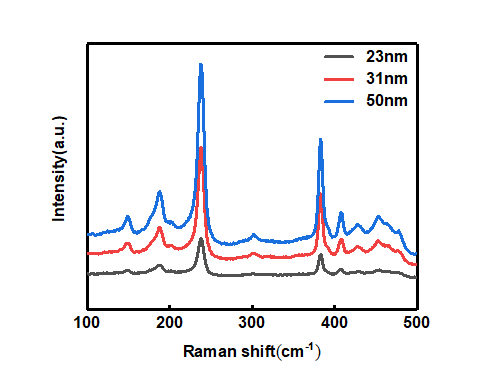


Figure S3: Raman spectra of different thicknesses

To analyze the relative content of the 1T phase and 2H phase in the samples, the characteristic peak J_1_ of the 1T phase and the characteristic peak E^1^_2g_ of the 2H phase in the Raman spectrum were selected as the objects for quantitative analysis, and the peak areas of the two were respectively integrated and calculated. Among them, the integral intensities of the J_1_ peak (I_1T_) of the three samples 23nm, 31nm, and 50nm were 47090, 146466, and 233482 respectively, and the integral intensities of the E^1^_2g_ peak (I_2H_) were 81693, 255405, and 408019 respectively. According to the quantitative formula (1T phase content = I_1T_ / I_2H_ × σ, where σ is the correction factor, with a value of 0.85) ^[1-2]^, it can be calculated that the 1T phase contents corresponding to 23nm, 31nm, and 50nm are 67.8%, 67.5%, and 67.3% respectively. The results show that the 1T/2H phase ratio in devices of different thicknesses is highly similar. This phenomenon further confirms that the 1T phase content is not the key factor determining the device response mechanism.


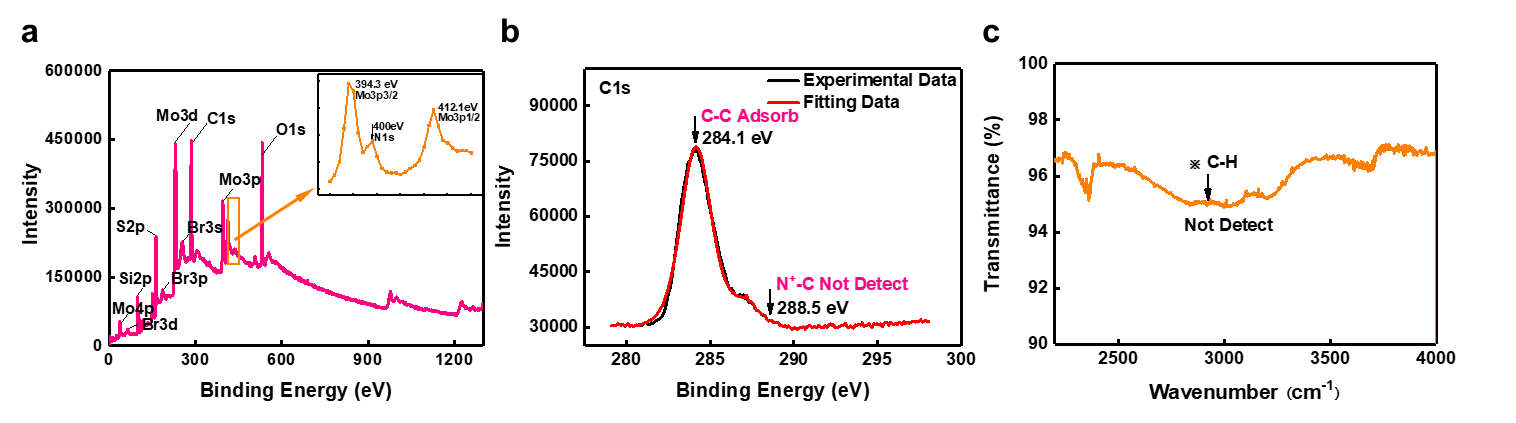


Figure S4: (a)X-ray Photoelectron Spectroscopy (XPS) patterns (b) C1s spectrum (c) FTIR analysis.

To verify the complete removal of the (C_7_H_15_)_4_N^+^ intercalant after the cleaning process, Fourier-transform infrared spectroscopy (FTIR) and XPS analyses were performed. The FTIR spectrum of the final film lacks all characteristic absorption peaks of the organic cation. Consistently, the XPS C1s spectrum shows no features beyond common adventitious carbon, and the survey scan detects no significant nitrogen signal. The negligible trace in the N1s region (inset of Figure S4a) is orders of magnitude too weak to correspond to residual intercalant and likely stems from instrumental background or ambient adsorption. These results confirm that the film's properties are intrinsic to the MoS_2_ phases.


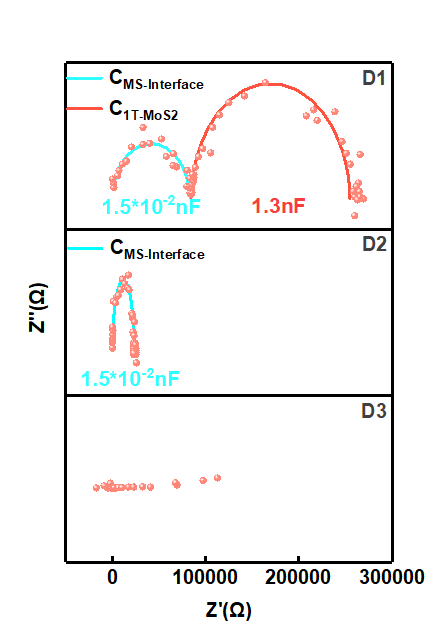


Figure S5 Impedance spectra of D1 D2 and D3 devices


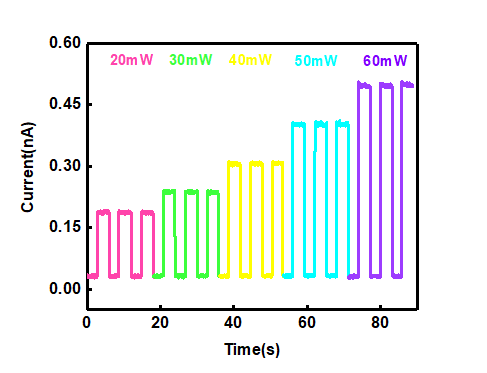


Figure S6 I-T curve of D2 device under 808 nm laser irradiation with different powers

Under 808 nm (20-60 mW) laser excitation, which delivered orders of magnitude higher thermal input to the device, the response transitioned from a spiking pattern to a typical rectangular photoconductive signal, and the spikes completely vanished, which eliminated the possibility of pyroelectric effect.


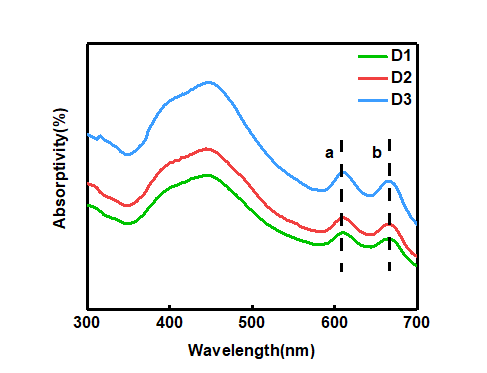


Figure S7 Absorption spectra of different thicknesses

All the samples show characteristic absorption peaks at 610 nm and 666 nm (peaks a and b), which originate from the direct band gap property and spin-orbit coupling effect of MoS_2_, respectively. As the film thickness increases from 23 nm to 50 nm, the intensity of the a/b peaks gradually enhances, and their light absorption ability is significantly enhanced. This implies that thicker nanosheets can absorb more photons under the same light condition, which in turn generates larger photogenerated voltage and photocurrent signals.

**Table S1:**

| Reference | This work | [3] | [4] | [5] | [6] | [7] | [8] | OS；optoelectronic synapse. |
| --- | --- | --- | --- | --- | --- | --- | --- | --- |
| Power supply | Self-powered and bias voltage | Self-powered and bias voltage | Self-powered and bias voltage | Bias voltage | Bias voltage | Bias voltage | Self-powered |  |
| Photoresponsivity (AW^-1^) | 0.0156 | 0.9 | 0.3 | 0.7 | 606.3 | 880 | 0.01 |  |
| Energy utilization efficiency | 87.6% |  |  |  |  |  |  |  |
| Response speed | 19.2ms | 72μs | 188μs | 60ms | 0.35s | 4s |  |  |
| Response mode | PC/PV/PCC | PV/PC | PV/OS | PC | PC | PC | PV |  |
| Operating modes count | Three | Two | Two | One | One | One | One |  |
| Photodetector (material) | MoS_2_ | Gr/MoS_2_/VO_2_ | MoTe_2_/MoS_2_ | MoS_2_/g-C_3_N_4_ | MoS_2_/N | MoS_2_ | MoS_2_/WSe_2_ |  |

**Reference：**

1. C.D. Wagner, J.F. Moulder, W.F. Stickle, P.E. Sobol, Atomic Sensitivity Factors for X-ray Photoelectron Spectroscopy, Surf. Interface Anal. 1981, 3, 211–222.
2. Moulder JF, Stickle WF, Sobol PE, Bomben KD. Handbook of X-ray Photoelectron Spectroscopy: A Reference Book of Standard Spectra for Identification and Interpretation of XPS Data. Edited by Chastain J. Eden Prairie, MinnCSOla: Perkin-Elmer Corporation Physical Electronics Division; 1992.
3. J. Wu, B. Guo, Z. Wen, Y. Wu, H. Wang, L. Wu, C. Wang, R. Che, Dual-mode photoresponse of the Gr/MoS_2_/VO_2_ heterostructure toward multifunctional optoelectronic applications ACS Appl. Electron. Mater. 2025, 7, 7345-7353.
4. Y. Ouyang, C. Zhang, J. Wang, Z. Guo, Z. Wang, M. Dong, Gate-tunable dual-mode optoelectronic device for self-powered photodetector and optoelectronic synapse Adv. Sci. 2025, 12, 2416259.
5. D.B. Velusamy, M.A. Haque, M.R. Parida, F. Zhang, T. Wu, O.F. Mohammed, H.N. Alshareef, 2D Organic–Inorganic Hybrid Thin Films for Flexible UV–Visible Photodetectors, Adv. Funct. Mater. 2017, 27, 1605554.
6. G. Polumati, C.S.R. Kolli, A. de Luna Bugallo, P. Sahatiya, Engineering surface state density of monolayer CVD grown 2D MoS₂ for enhanced photodetector performance, PLoS ONE 2024, 19(4), e0297825.
7. O. Lopez-Sanchez, D. Lembke, M. Kayci, A. Radenovic, A. Kis, Ultrasensitive photodetectors based on monolayer MoS₂, Nat. Nanotechnol. 2013, 8, 497–501.
8. C.-H. Lee, G.-H. Lee, A.M. van der Zande, W. Chen, Y. Li, M. Han, X. Cui, G. Arefe, C. Nuckolls, T.F. Heinz, J. Guo, J. Hone, P. Kim, Atomically thin p–n junctions with van der Waals heterointerfaces, Nat. Nanotechnol. 2014, 9, 676–681.
